# Supplementary material for: Sorting out assortativity: When can we assess the contributions of different population groups to epidemic transmission?
Source: PLoS One. 2024 Dec 2;19(12):e0313037. doi: 10.1371/journal.pone.0313037 (PMC11611214; doi:10.1371/journal.pone.0313037)
Supplement: Attachment — Submitted filename: Review Response.docx [file pone.0313037.s002.docx]

Dear Mr. Geismar,

Thank you very much for submitting your manuscript "Sorting out assortativity: when can we assess the contributions of different population groups to epidemic transmission?" (PCOMPBIOL-D-24-00352) for consideration at PLOS Computational Biology. As with all papers peer reviewed by the journal, your manuscript was reviewed by members of the editorial board and by several independent peer reviewers. Based on the reports, we regret to inform you that we will not be pursuing this manuscript for publication at PLOS Computational Biology.

Associate editor

The paper has been read by two experts in the field and by myself in less detail. We find the problem of potential interest. But at the same time the manuscript does not hold high enough quality to be published in PLoS CB. The referees raises several concerns that I agree with. In particular there is too little novelty in methodology. It seems more to present some rule of thumbs regarding assortativity and it is unclear what these should be used for. For details I refer to the comments of the two referees. Please note that this by no means imply that the paper is wrong or uninteresting, but simply that it is not suitable for PLoS CB given its high submission pressure.

Kind regards, Tom Britton

The reviews are attached below this email, and we hope you will find them helpful if you decide to revise the manuscript for submission elsewhere. We are sorry that we cannot be more positive on this occasion. We very much appreciate your wish to present your work in one of PLOS's Open Access publications.

Thank you for your support, and we hope that you will consider PLOS Computational Biology for other submissions in the future.

Sincerely,

Tom Britton

Academic Editor

PLOS Computational Biology

Thomas Leitner

Section Editor

PLOS Computational Biology

**Reviewer #1:**

1. This is an interesting paper that presents a new method for estimating assortativity in disease transmission given full knowledge of the transmission network. While the authors acknowledge that such full knowledge is rarely available in practice, and that the specific model for assortativity is somewhat crude, the paper sets a baseline for developing more advanced methods that account for partial and/or uncertain knowledge of transmission networks. As such, it is an important advance already, and others will surely build on it.

Thank you for recognising our paper as an important advance in estimating transmission assortativity. Your feedback on potential future developments building upon our method is greatly encouraging and we are currently working on adapting this method to uncertain knowledge of transmission networks.

2. My major concern with the paper is that it seems to ascribe special significance to the transmission peak in its evaluation of this method's accuracy (e.g., the last sentence of the abstract, the dependence of the results in Fig 1C on the "peak coefficient", etc.). However, I suspect that this observation is not causal; instead, it is more coincidental that an estimator based on a ratio of event counts (Eqs (5) and (4)) is very noisy for low counts and only sharpens to something useful when the counts rise to the 10s to 100s. In the particular scenarios explored in the simulations (from the SI, group sizes are uniform in the range 20-200, with a handful of groups per simulation), the above condition seems to coincide with the transmission peak, but that coincidence need not be present other scenarios or in a real-world outbreak. I would strongly urge the authors to explore a radically different scenario where such a coincidence is absent, e.g., with slower growth (say R0 just above 1) in a much larger population (say 1M); given the experience of SARS-CoV-2, it's clear that such large outbreaks are not a mere academic fancy. My sense is that their estimator would start to become quite sensitive and unbiased once several 100 transmissions have taken place, regardless of how far into the outbreak this occurs. Moreover, if that point is hit early in the outbreak, the assumption of the population being almost completely susceptible is still mostly true, so the estimator will be more accurate than near the transmission peak.

Thank you for your feedback. We agree that our estimator's performance is primarily linked to the number of observed cases before significant group saturation, rather than the epidemic peak itself. We have revised the results to clarify that the estimator's reliability improves with increasing case numbers, typically requiring at least 30 observations per group before saturation occurs. We now also explain that our guideline referencing the peak timing is based on the hypothesis that early in an outbreak, up to the peak, the depletion of the susceptible population is not substantial enough to notably alter transmission dynamics. This hypothesis is now explicitly stated in new text added to the introduction introduction and methods:

*“Furthermore, existing methods often fail to account for the rapid depletion of susceptibles typical in small outbreaks, such as those in healthcare settings. This saturation effect can significantly influence transmission patterns, as a saturated group cannot sustain further transmission within the group. Thus analysing proportions of transmission types across an entire outbreak, beyond the point of saturation, may not accurately reflect the underlying baseline transmission patterns.”* Introduction section, page 6.

*“We evaluated the estimator's performance at various stages of the outbreak, defined in relation to the group's epidemic peak, i.e. the day with the highest symptom onset incidence following the first case. We hypothesise that in the early stages of an outbreak, up to the group’s epidemic peak, the depletion of susceptibles is not substantial enough to significantly alter transmission dynamics.”* Methods section, page 12.

We have also modified the introduction to better contextualise our work, which was motivated by previous research using outbreak reconstruction tools to analyse nosocomial respiratory outbreaks, characterised by small group sizes, where saturation occurs rapidly. This focus informed our simulation design, which explored a wide array of small outbreak scenarios using a range of R0 values (mean = 3, 95% quantile: 1.09 - 6.25), including scenarios just above R0 = 1 (see supplementary material Figure S2).

While we appreciate the suggestion to explore larger population scenarios, our study's scope aligns with the practical limitations of outbreak reconstruction tools that require extensive data collection generally only available in small settings, and which can typically only handle a limited number of cases. We believe our edits to the introduction clarify the background, motivation, and intended application of our work within the context of small-scale, particularly nosocomial, outbreaks .

*“Healthcare settings are particularly suited for employing advanced outbreak reconstruction tools due to the relatively small size of nosocomial outbreaks, regular surveillance and data collection, and access to whole genome sequencing [20–22]. Such tools typically leverage pathogen genetic sequence data, symptom onset or test collection dates, and contact data within a Bayesian framework to probabilistically reconstruct transmission events, generating posterior sets of transmission chains [23–26].”* Introduction, page 5.

3. More ambitiously, it should be possible to adapt their framework to something like a continuous SEIR model (or maybe something simpler), where the only variables are the fractions of each group in each of the SEIR compartments through time (i.e., something like an "infinite population" limit). There is then a time-dependent analog of Eq (5) for the estimated fraction of secondary cases in group "a" at time t caused by infected individuals in group "a" at some time t - delta t, where the numerator and denominator on the right-hand side of Eq (5) turn into derivatives of some of the model quantities. This would (a) make clear that pi_{a <- a} can be estimated using only the transmissions shortly before/after any particular time t, regardless of where that lies w.r.t. the transmission peak, if only there were enough such transmissions for counting to be effective; and (b) possibly describe analytically the exact way in which the gradual saturation of the population distorts the point estimate of assortativity. The idea in this paragraph would, I think, add significantly to the paper, but is a suggestion, not a request for the review.

Thank you for your suggestion. While we agree that this is an interesting research avenue which merits exploration in future research, adapting our framework to a continuous SEIR model exceeds the scope of our current study.

4. A second important concern that does need addressing is in the presentation. I feel that the authors might know their model too well at this point to remember what it's like to see this model for the very first time, and some important details and definitions are absent or only hinted at. For example, in Eq (7), the function w(t) isn't defined anywhere, but divining from the descriptions in the SI, it seems to be some discretized convolution of the Gamma distributions for the incubation and generation times (?). Similarly, in the description of the Methods (lines 132-141), it's not at all clear initially that beta_{b <- a} is the same for every b distinct from a, but may depend on a: the offending equation is "beta_{a <- a} = gamma_a beta_{b <- a} (with a != b)". More subtly, it took me some time to understand that the transmission rate beta_{b <- a} doesn't include an explicit dependence on the total population size, so that naively, if I grow the population size, the total rate at which transmissions takes place grows too. This is later counteracted in Eq (7), where only ratios of transmission rates enter into pi_{b <- a} and the number of transmissions is controlled instead by R_{0a} (it hurts my head to convince myself that the setup of the beta_{b <- a} rates is consistent with having different R_{0a}'s for each group a, but in the end, I think it's related to beta_{b <- a} (b != a) being different for each a; is this true?). Mysteriously, this *does* involves scaling the force of infection in (9) by the population size N_b (somehow, lamba^j_{b <- a}(t) was "the force of infection [...] towards each individual in group b" (line 174), but after adding over all possible individuals j from all other groups a, the total becomes "the total FOI that *group b* receives" (line 179, my emphasis), which presumably motivates the division by N_b in Eq (9): what gives?). In the end, I think I (just about) get the overall picture of what's going on, but feel that the authors could set up the scenario and the resulting calculations in a way that's far more straightforward for someone to understand when first presented with this model. Could it be as simple as polishing the descriptions and also defining beta_{b <- a} = nu R_{0a} / N for some appropriate rate nu ? A final remark: the simple derivation in Sec 1.1 of the SI is so short and so key to the main part of the paper that it would be better to put it in the main text.

Thank you for your feedback. We agree that the description of the methods was not clear enough - a point also noted by the other reviewer.

As per both reviewers’ suggestions, we have modified the manuscript to improve the clarity and accessibility of our methods. Here is a summary of the key changes:

- Definition of *w*: We have now explicitly defined *w* in the ‘Simulation study’ section on page 10 as the probability mass function of the generation time distribution. “*w is the probability mass function of the generation time distribution”,* page 10.
- Clarification of β_b←a_: We have rewritten the description to clearly state that 1) β_b←a_ is the same for every *b* distinct from *a* and 2) it doesn't directly depend on total population size, as it is a person-to-person transmission rate. *“We make the following assumptions:*

*β_b←a_ is the same for all b ≠ a, i.e., β_b←a_ = ψ if a ≠ b.*

*β_a←a_ = γ_a_ψ, where γa is the assortativity coefficient for group a.”* Methods section, page 7.

- Supplementary Information: As suggested, we have moved the derivation from Section 1.1 of the supplementary material to the methods section (page 8). *“We derive π_b←a_ , the proportion of secondary cases in group b amongst those generated by infectious individual in group a, where a ≠ b , as:*

|  | *(2)* |
| --- | --- |

*We derive π_a←a_, the proportion of secondary cases in group a amongst those generated by an infectious individual from the same group, as:*

|  | *(3)* |
| --- | --- |

*We can obtain γ_a_ by rewriting equation 3 as:*

|  | *(4)* |
| --- | --- |

- Force of infection scaling: Following equation 8, we have now clarified how the force of infection is calculated and scaled from individual to group level, clarifying the need for division by *N_b_* in Equation 9.

*“The total FOI that all individuals in group b collectively receive from all individuals across all groups at time t is obtained as:*

|  | *(8)* |
| --- | --- |

*Hence, the FOI that one individual from group b is exposed to is**.”* Simulation study section, page 10.

We believe these changes significantly improve the clarity of our methods.

5. Apart from the two significant points above, the rest of the simulation & analysis work seems thorough and well-done to my eyes. Anyone using this estimator in the future will find the characterizations of its performance here very useful and comprehensive. I don't see any pressing need to revise these significantly (after accounting for "peak" vs "enough counts" above).

Thank you for your positive feedback. We have acknowledged and addressed the point regarding "peak" versus "enough counts" (see answer to point 2.).

**Reviewer #2:**

1. This paper provides a tool to estimate group-to-group transmission patterns and assortativity from transmission tree data, accounting for group size. However there are some strongly limiting assumptions, in particular that all groups have the same person-to-person transmission rate to group a except group a itself, and that transmission chains are known perfectly.

We acknowledge that assuming a uniform person-to-person transmission rate (β_b←a_) across all groups except group *a* itself is a strong and somewhat limiting assumption. However, this is a common assumption [[1–3]](https://www.zotero.org/google-docs/?PwRF2N), and this limitation only applies in the case where there are more than two groups. We are now acknowledging this limitation in a new dedicated paragraph in the discussion section, where we also highlight studies showing that such models based on assortativity can effectively mimic contact and transmission patterns across diverse contexts.

*“Characterising transmission through assortativity implies that a group's transmission patterns are identical towards all other groups, with only the within-group pattern being distinct. While this approach is fully representative in a two-group scenario, it is limiting when additional groups are involved. Nevertheless, this simplification aligns with established research on social networks and disease transmission dynamics suggesting that assortativity coefficients alone can effectively capture the essence of contact and transmission patterns across various contexts [27,28,35].”* Discussion section, page 18.

2. The study does not analyze data but the authors provide extensive simulations to test their method. The results are hard to interpret. But I appreciate that the method is simple and accessible. The authors comment that a major limitation is the assumption that transmission trees are known. Indeed, probably the reason they don’t include a data analysis is that transmission trees are very rarely known. This makes me wonder how applicable this method is.

We appreciate the reviewer's feedback. While we do acknowledge that transmission trees are never exactly known, our research was prompted by the growing accessibility of tools to probabilistically reconstruct transmission chains, thanks in particular to advancements in genome sequencing. Thanks to these approaches, it is now often possible to obtain a posterior sample of plausible transmission trees, each of which could be analysed with the method we propose, with assortativity estimates integrated across all plausible transmission trees. We have revised the introduction to more clearly position our work within the context of previous studies that used these tools to examine nosocomial transmission patterns and their associated challenges.

*“Healthcare settings are particularly suited for employing advanced outbreak reconstruction tools due to the relatively small size of nosocomial outbreaks, regular surveillance and data collection, and access to whole genome sequencing [20–22]. Such tools typically leverage pathogen genetic sequence data, symptom onset or test collection dates, and contact data within a Bayesian framework to probabilistically reconstruct transmission events, generating posterior sets of transmission chains [23–26].”,* page 5.

“*Our approach quantifies group-specific transmission assortativity, from known or probabilistically reconstructed transmission chains, while accounting for the relative sizes of each group in the susceptible population.”,* page 6.

*“Nevertheless, our approach is adaptable and can be extended to reconstructed transmission chains, for example, by estimating the assortativity coefficient across all posterior transmission trees. Future research should delve into understanding how uncertainty surrounding these transmission trees further impacts our ability to infer transmission patterns.”,* page 17.

3. Below equation 5 - the authors observe transmission pairs and these go into the inference somehow in an estimate of delta (\hat{delta}). We need an explicit definition of \hat{delta} and how it’s computed. This should be earlier, in the methods section about the inference, not about the simulator. As it is, the simulations and inference parts are a bit conflated. Since after (6) you the authors note that all results are presented using delta rather than gamma, they should state the math in terms of delta (and then describe the estimator \hat{delta}). They also need to state explicitly what data from the simulations is used in the estimation.

We appreciate the reviewer's valuable feedback. In response, we added the explicit definition of delta in the manuscript in equation 6 and provided a detailed explanation of how delta hat is computed in supplementary material. In brief, the uncertainty in delta is drawn from a binomial process around the proportion of within group transmission (*τ_a←a_*/*τ_.←a_*). Additionally, we have explicitly outlined in the methods section that the estimation process of delta relies on two inputs: 1) the number of transmissions pairs observed between any two groups a and b, i.e. where the infector is in group a and the infected in group b (tau_b<-a), and 2) the relative sizes of the groups (f_a). In addition, we use the dates of symptom onset to restrict the inference to pairs where both infectors and infected individuals have had symptoms before a threshold date *T*.

*"The proportion of within-group transmission, π_a←a_ (equation 3), can be directly calculated from known transmission chain data. It is calculated by dividing the number of observed within-group transmission pairs, τ_a←a_, by the total number of transmissions originating from group a, τ_.←a_ .Thus the proportion, π_a←a_ = τ_a←a_/τ_.←a_ , ranges between 0 and 1 (included).*

*To simplify interpretation, we introduce a rescaled parameter δ, ranging between -1 (fully disassortative) and 1 (fully assortative), with 0 corresponding to a homogeneous transmission pattern (Figure S1) such that:*

|  | (5) |
| --- | --- |

*The formula for δ_a_ can thus be written as:*

|  | (6) |
| --- | --- |

*where:*

- *τ_a←a_: represents the number of transmissions from group a towards group a.*
- *τ_.←a_: refers to the total number of transmissions emitted by group a.*
- *τ_a←a_/τ_.←a_: Denotes the proportion of within-group transmissions for group a denoted as π_a←a_ in equation 3.*
- *f_a_: Represents the proportion of the total population that belongs to group a. It is a value between 0 and 1, exclusive of the endpoints as there must be more than 1 group in the population.”* Methods section, page 8-9.

We clarified in the manuscript what data was produced by the simulation and how it was used to estimate delta:

*“The simulation outputs a transmission tree that includes, for each infected individual, their symptom onset date, their group affiliation, and the id of their infector. Using this data for all infected individuals with symptoms up to time t, we calculated τ_a←a_ (the number of within-group transmission pairs) and τ_.←a_ (the total number of transmissions originating from each group). These values, along with the relative sizes of the groups (f_a_), were input into equation 6 to estimate the assortativity coefficients for each group”*. Simulation study section, page 10.

We clarified in the supplementary material, section 1, how the central estimate, upper and lower bound for delta were obtained.

*“1.	Accounting for the uncertainty in δ_a_ through π_a←a_.*

*Using transmission chain data, we obtain the observed proportion of within group transmission, π_a←a_ (equation 3 of the manuscript), by dividing the number of observed within-group transmission pairs, τ_a←a_, by the total number of transmissions originating from group a, τ_.←a_ .*

*Confidence intervals around that mean estimate can be obtained using the Clopper-Pearson binomial interval method* [*[1]*](https://www.zotero.org/google-docs/?BSAzxK)*, as:*

*where:*

- *π_a←a lb_ is the lower bound of the CI for π_a←a_.*
- *π_a←a ub_ is the upper bound of the CI for π_a←a_.*
- *Beta is the quantile function of the beta distribution.*
- *p is the probability value.*
- *α is the significance level.*
- *s1 and s2 are the shape parameters of the Beta distribution.*
- *τ_a←a_ is the number of within-group transmissions in group a.*
- *τ_.←a_ is the total number of transmissions emitted from group a.*

*Note that the relationship between δ_a_ and π_a←a_ in equation 6 of the manuscript, is monotonic and hence preserves confidence intervals. Therefore central estimates and confidence intervals for δ_a_ can be obtained by substituting the central estimate and confidence interval bounds for π_a←a_ into the formula for δ_a_ (equation 6 of the manuscript), as follows:*

*”*

Supplementary material, Section 1, page 2.

4. It’s surprising that the authors look at the peak of the epidemic but don’t include different Sb (susceptible population for group b) sizes in the equations for the fractions of infection, pi, from group b to group a. Why don’t you need to take the susceptible size into account, not the total group size? (I suspect that you do, and this could be why the estimates are not better, but it’s hard to tell because we don’t hear what data are used from the simulations or exactly how)

We thank the reviewer for their comment. We have clarified in the introduction the motivation and background for our work. Our study focuses on person-to-person transmitted pathogens within hospital settings, where we typically encounter completely susceptible populations at the outbreak's onset. This context justifies our approach of not explicitly including different sizes of susceptibles in the equations for *π (equations 2 and 3)*.

*“We evaluate the performance of our estimator through diverse simulations, mimicking nosocomial outbreaks, where the populations are fully susceptible at the outbreak’s onset.”*Introductions section, page 6.

*“We simulated small outbreaks under various contexts to assess the estimator’s performance in scenarios relevant to person-to-person transmission of healthcare-acquired pathogens in a fully susceptible population”* Simulation study section, page 9.

5. PLOS CB is a methods-positive journal with a theory-savvy readership. I think more methods can be in the main text, including parameters for the simulation. We must have more about the regression - why do it, what does it mean, what are the variables considered, etc? We get to section 1.5 of the supplement with only the slightest mention of this in the Results of the main text and none in the methods.

We have enhanced the description of the estimator's formula and simulation methodology based on reviewer suggestions, moving section S1.1 of the supplement into the main text. If the editors of PLOS CB are willing to reconsider their decision, and should they agree that it is desirable to move more methods into the main text, we would be happy to do so.

6. Why would gamma be infinity?

We explained that gamma = infinity corresponds to full assortativity, which means that individuals in a group only transmit the infection to others within the same group. Gamma measures the excess probability of transmission within-group compared to between-group. In the model, gamma equals infinity when the proportion of transmissions occurring within the same group (π_a←a_) equals 1, implying that the proportion of between-group transmissions π_b←a_ equals 0 (see equation 4).

We added a more explicit description of full assortativity and full disassortativity in the methods definition page 7:
“γ values range from 0 (fully disassortative, i.e. no within-group transmissions) to ∞ (fully assortative, i.e. transmissions occur exclusively within the group), with 1 indicating homogeneous patterns.” methods, page 7.

We also added a paragraph to clarify the range of *π* in the methods: *“Thus the proportion, π_a←a_ = τ_a←a_/τ_.←a_ , ranges between 0 and 1 (included).” methods, page 8.* It should be clear from equation 4 that when *π_a←a_* = 1, gamma is infinity.

7. This work also conflates transmission and contact, which is understandable since with case data we can only really see the product of the two (but it should be mentioned).

Thank you for your suggestion, we have added this clarification in the introduction:
*“These group dynamics are not only determined by distinct contact patterns, as revealed by large-scale contact surveys studies, such as POLYMOD* [*[6]*](https://www.zotero.org/google-docs/?ENrnrI) *and CoMix* [*[7]*](https://www.zotero.org/google-docs/?iCAh0D)*, but also by varying infectiousness and susceptibility levels* [*[1,8–11]*](https://www.zotero.org/google-docs/?UYqSul)*.”* Introduction, page 4.

This clarification was already present in the methods:

*“The resulting transmission patterns thus reflect not only mixing patterns but also differences in infectiousness and susceptibility amongst groups.”, methods page 7.*

*We have also now clarified that we estimate “transmission assortativity” throughout the text.*

8. The authors should look at Kadelka 2023 for a definition of homophily of a contact matrix that has the advantage that it ranges from -1 to 1, for entirely dissortative mixing to completely assortative (a disconnected graph). <https://pubmed.ncbi.nlm.nih.gov/36899581/> is Kadelka’s paper.

Thank you for highlighting Kadelka's work.

Their approach and our parameter delta both yield metrics ranging from -1 to 1 for disassortative to assortative patterns. However, our research differs significantly in scope and application. Indeed we define an assortativity metric specific to each group, whilst they define an homophily metric at the whole population level. Therefore their homophily metric alone would not enable identification of specific groups overwhelmingly contributing to transmission and hence is not directly relevant to our work.

9. In equation (9) why divide by Nb? I think it is because lambda_b in (8) is the entire FOI that group b receives so each member of b only receives 1/Nb of it. So the probability that an individual k in group b is not infected in the time step is the product (over all the potential infectors in all the groups) that the infector does not infect k. If so, please explain.

Yes this is correct and we have clarified the explanation in the main text below equation 8.

“*The total FOI that all individuals in group b collectively receive from all individuals across all groups at time t is obtained as:*

|  | *(8)* |
| --- | --- |

*Hence, the FOI that one individual from group b is exposed to is**.”*page 10.

10. What happens if the model is mis-specified? This is hard to envision because we don’t know what data from the simulations the authors used and how.

1 - We have clarified what data is used and how (see answer to point 3).

2 - *δ*_a_ only relies on *f*_a_ and *π_a←a_* (see equation 4). We didn't formally assess the performance of our model on data generated with a transmission matrix which deviates from our assumption of uniform transmission outside of the group. However, as highlighted in the discussion, this only affects inference beyond 2 groups, and given that only *π_a←a_* is used in the estimator, we do not expect the structure of the off-diagonal of that transmission matrix to significantly affect our results. However, we do expect misspecification of *f_a_* to affect the estimate. We have now added a heatmap which illustrates this effect (see supplementary figure S1.2) and added a reference to that figure in the methods:
*“The relationship between δ_a_, π_a←a_ (i.e. τ_a←a_/τ_.←a_), and f_a_ can is visually represented in supplementary figure S1.2”* Methods, page 9.

*“Another limitation of our approach includes that our estimator requires, and is quite sensitive to (figure S1.2), information on group sizes which may be difficult to obtain in real-life settings, however various methods exist for population size estimation* [*[34]*](https://www.zotero.org/google-docs/?2xHZ2U)*.”* Discussion section, page 18.

11. Finally, it’s hard to imagine that we could know the transmission chains perfectly and still not be able to estimate the group-to-group transmission dynamics. Where we can’t, why not?

Even with known transmission chains our work highlights how several factors complicate the estimation of group-to-group transmission dynamics:

1. Saturation effects: As the epidemic progresses, the depletion of susceptible individuals alters the underlying baseline transmission patterns. If data from late in the outbreak, past the group's peak, is used for inference, it may lead to misestimation of the baseline group transmission dynamics and conflate them with group-specific immunity.
2. Interdependence of group dynamics: Transmission patterns and saturation in one group can indirectly affect other groups.
3. Small sample sizes: Our study simulated small epidemics to resemble that of nosocomial outbreaks. The small sample size leads to high uncertainty in estimates.

These factors underscore the importance of careful interpretation of transmission dynamics estimates in small outbreak settings, even with complete data. We have significantly edited the introduction section to explain the motivation for our work and the challenges posed by small outbreaks when assessing group dynamics:

*“Furthermore, existing methods often fail to account for the rapid depletion of susceptibles typical in small outbreaks, such as those in healthcare settings. This saturation effect can significantly influence transmission patterns, as a saturated group cannot sustain further transmission within the group. Thus analysing proportions of transmission types across an entire outbreak, beyond the point of saturation, may not accurately reflect the underlying baseline transmission patterns.”*

12. The authors could look at their transmission chains and see if the pi_{b <-a} fractions are what their math says they should be; if not why not, etc?

Thank you for your suggestion. We have now added a model validation section in the supplementary material (section 4). We simulated a "null" scenario with five identical groups, each having neutral assortativity (δ = 0). Our mathematical framework (equations 2 and 3) predicts that the observed proportions of within-group (π_a←a_) and between-group (π_b←a_) transmissions should be equal to ⅕ . The simulation results, shown in a new figure (Figure S7), align with this prediction.

**References**

[[1]	Newman MEJ. Assortative Mixing in Networks. Phys Rev Lett 2002;89:208701. https://doi.org/10.1103/PhysRevLett.89.208701.](https://www.zotero.org/google-docs/?JVdHdP)

[[2]	Nishiura H, Cook AR, Cowling BJ. Assortativity and the Probability of Epidemic Extinction: A Case Study of Pandemic Influenza A (H1N1-2009). Interdiscip Perspect Infect Dis 2011;2011:194507. https://doi.org/10.1155/2011/194507.](https://www.zotero.org/google-docs/?JVdHdP)

[[3]	Badham J, Stocker R. The impact of network clustering and assortativity on epidemic behaviour. Theor Popul Biol 2010;77:71–5. https://doi.org/10.1016/j.tpb.2009.11.003.](https://www.zotero.org/google-docs/?JVdHdP)
